# Section on Ophthalmology, College of Physicians of Philadelphia

**Published:** 1897-02

**Authors:** Howard F. Hansell

**Affiliations:** Clerk of Section; Philadelphia


### SOCIETY REPORTS.

---

### SECTION ON OPHTHALMOLOGY, COLLEGE OF PHYSICIANS OF PHILADELPHIA.

PHILADELPHIA, November 17, 1896.

Dr. J. M. Da Costa in the chair.

"Unilateral Albuminuric Retinitis," with a case, by G. E. de Schweinitz, M.D. After a brief review of the literature of unilateral albuminuric retinitis, during which reference was made to fifteen cases, Dr. de Schweinitz reported two examples that had come under his care, both in colored men. In the one instance clinical examination indicated chronic nephritis, and there was unilateral neuro-retinitis. The patient, however, was not seen again and his subsequent history was unknown. In the second case the patient had been under observation for five months, and had all the symptoms of chronic interstitial nephritis with unilateral (right side) retinal lesions. Water colors illustrating the condition in two stages, were presented, indicating that the primary lesion had probably been a thrombosis in the lower nasal vein with secondary involvement of the disc and retina. Dr. de Schweinitz agreed with Knies that unilateral albuminuric retinitis is not so great a rarity as some text-books would lead us to believe. A certain percentage of cases maintain monocular retinal lesions until death; in another the unilateral character of the affection is maintained for a considerable portion of time, but ultimately becomes bilateral. Dr. de Schweinitz suggested that an interesting clinical observation in these unilateral cases would result from catheterization of the ureters and separate analysis of the urine from each kidney. His patient had declined to submit to this procedure.

"Report of the Successful Removal of a Piece of Steel from the Vitreous by the Hirschberg Magnet," and exhibition of the patient, by G. Oram Ring, M.D. J. S., aged twenty-eight, was admitted to the wards of the Episcopal Hospital, June, 1896. Six hours previously a piece of steel from an anvil penetrated the right lower lid and the ocular coats, and was lodged in the nasal side of the anterior portion of the vitreous, where its position could be distinctly outlined. The cornea, iris, and lens were uninjured. A minute bead of vitreous protruded from the wound in the sclera. The choroid was ruptured and retinal hemorrhage was profuse at the site of the injury, V. $\frac{20}{200}$. The following day, under antisepsis and cocaine anesthesia, an incision was made with a Graefe knife through the conjunctiva and sclera six mm. from the corneal border and opposite its lower third. While the edges were retraced by an assistant, the straight tip of a Hirschberg magnet was inserted. Upon withdrawal of the magnet, after its second introduction, a piece of steel 6 x 2 x 1 mm. was found clinging to it. No sutures were used. Recovery was prompt and uneventful. A patch of atrophied choroid corresponding to the rupture can be seen through the now transparent vitreous. The vision has increased to $\frac{20}{70}$.

"A Case of Foreign Body in the Vitreous," with exhibition of the patient, by M. W. Zimmerman, M.D. The patient, J. J., was wounded by the explosion of a copper dynamite cartridge January 18, 1890. The fragment entered the sclera of the left eye seven to eight mm. to the nasal side of the corneal limbus. Blood filled the anterior chamber for two days. After absorption the foreign body could be seen in the vitreous opposite the base of the iris on the temporal side. A drawing made at this time represents exactly its present position and appearance. One year later there was a moderate hyalitis of unknown origin, and confined to this eye, ending in complete recovery. The patient was treated at this time by the late Dr. George T. Lewis, whose notes furnish the above facts. The boy consulted me first in March, 1896, on account of accommodative asthenopia. A weak hyperopic cylinder relieved the symptoms and gave normal vision, which has continued. The presence and unaltered position of a piece of copper for seven years without irritation give interest to the case, and particularly in view of the opinion of Leber and others, that copper is more dangerous to the safety of the eye than other metals. In this case the fragment has become lightly encysted and gives no metallic reflex, excepting in a very dark room, where it emits to the illumination of the ophthalmoscopic mirror a reddish tinge.

Dr. H. F. Hansell read a paper upon "Report of the Successful Removal of a Piece of Steel from the Vitreous by the Hirschberg Magnet," and exhibited the patient. J. S. received a small fragment of metal in the left eye in April, 1896. After the transient discomfort had subsided, he gave the accident no further thought, until it was revived in his memory by the questions asked when he applied in October at the Jefferson Hospital, on account of failing vision and inflammation in the eye. A bright reflecting piece of metal could be readily seen with the ophthalmoscope floating in the vitreous, and a small scar below, and to the outside of the corneal limbus was found V. $\frac{20}{200}$. An incision through the conjunctiva and sclera between the external and inferior rectus was made, through which the smallest tip of the Hirschberg magnet was inserted. After two unsuccessful efforts, a small triangular, corroded piece of steel was removed with the loss of an insignificant amount of vitreous. In one week the vision was $\frac{20}{50}$. Three points may be noticed in connection with this case; namely, the comparatively long time between the entrance of the steel and its retraction of the edges of the scleral cut as the tip of the magnet was withdrawn, and the recovery of excellent vision.

Dr. Charles A. Oliver presented a "Brief Clinical and Histologic Study of a Case of Epithelioma of the Corneo-Scleral Junction." The condition was found in a sixty-nine-year-old man. The growth first manifested itself as a small "pimple" at the lower outer corneal border of the right eye, and gradually and painlessly increased in size. When first seen, it appeared as a fleshy and wart-like looking mass about the size of a pea, and embraced an area equal to almost the lower outer quadrant of the cornea.

In spite of careful excision with free thermo-cauterization repeated more extensively some two months later, the mass recurred until in four months' time it had become so great in size and so angry in appearance, that the eyeball with the surrounding conjunctiva was removed, the operation, by reason of renal and cardiac disease in a weak and feeble patient, being almost painlessly done during local anesthesia by the use of hydrochlorate of cocaine. Up to present writing there has not been any recurrence.

From a clinical standpoint the case is most interesting. Commencing as a "pimple" in the epithelial structures of the conjunctiva at the transition-border between the cornea and sclera, as is almost universal in such cases, the mass gradually and painlessly increased in size until it assumed the papillomatous variety of growth. It then extended into and beneath the epithelium of the cornea far in toward the summit of the membrane. In other words, the tumor mass evidenced its development and growth in a manner that is eminently characteristic of epitheliomatous formations.

The quick recurrence and steady increase of the growth, in defiance of the extreme radical measures employed for extirpation, manifestly evidenced the necessity of removal of the entire field of malignancy. The almost uncontrollable oozing of blood, experienced during the operative procedure, plainly showed the extreme vascularity of the neoplasm.

Microscopically, the specimen was exceedingly instructive, not only by reason of presenting the characteristic appearance of epitheliomatous formation in the region involved, but upon account of the undoubted protrusion of the epithelial cells into the interlamellar corneal spaces (which possibly might have been produced or rendered more easy by the operative procedures pursued in the earlier stages of the disease), and the insertion of the same form of malignant cells into the superficial layers of the sclera (layers which were untouched by operation); but is also of great interest in substantiating the view that the deepest penetrations of the epithelial cells into the outermost tunics of the eye were in the transition zone between the cornea and the sclera—that is, at the corneo-scleral junction.

Dr. Oliver exhibited a series of "Ophthalmoscopic Pictures of Peculiar and Rare Chorio-Retinal Changes, the Result of Traumatism." The first of this grouping was seen but a few hours after the patient, a man of forty-two years of age, had been struck in the left eye by a fist. Almost total blindness ensued immediately after the accident.

The eyeball was unruptured. The cornea seemed unusually brilliant. The anterior chamber was deepened, especially in its peripheral portion. The pupil was round, and the iris, though tremulous, was mobile to consensual reaction. The lens was dislocated directly back, its superior border resting against the retina just behind the inferior portion of the equator of the globe. The vitreous humor contained some rather fixed, doubtful streaks of blood in its anterior portion.

As shown in a water color sketch which was exhibited, the optic disc was greenish in tint and appeared bloodless. There were a few deeply situated hemorrhages in the retina, and a series of large choroidal ones extending along the retinal vessels, which were reduced to mere threads. These were broad and greenish elevated areas, as though the deeper retinal tissues against the choroid were thickened, swollen and opaque. The patient became blind in a few minutes and never regained vision.

In contrast with this sketch, which illustrated the grossest effects as seen ophthalmoscopically from concussion accidents, Dr. Oliver exhibited water color drawings of five other cases extending from minor degrees of visible change to the more pronounced varieties, one of which (the fourth example) closely resembled the chromo-lithograph in Jonathan Hutchinson, Jr.'s, well-known case.

Dr. Edward Jackson presented a case of "Deficiency of Pigment, Allowing the Fundus Reflex to Show through the Iris." At a former meeting of the Section he had shown a similar case. Both of these patients had undergone cataract extraction; in the one shown this evening there was rupture of the sphincter, the lens having proved to be larger than the average. It was the only time he had met this accident in simple extraction. The fundus reflex showed through other parts the iris almost as readily as through the part at which the rupture had occurred. Possibly it might be that there were other ruptures of the iris, but the distribution of the fundus reflex was entirely different from that indicating rupture of the iris. It constituted a general area of red against which the details of the iris were seen. It was rather a rounded area, and did not consist of fissures through which red reflex could be obtaine d It could hardly be regarded as a rupture of the posterior layer of the iris because there was no change in the outline of the pupil.

There was no deformity of the pupil in the case previously reported. Rupture of the posterior layer would necessarily cause deformity in the pupil, certainly a rupture of such considerable size. The posterior layer opposes the action of the sphincter, and the two together give the pupil its form. It is quite possible that this appearance is the result of atrophy of the pigment cells from stretching of the iris, but probably it is simply an atrophy comparable to the changes in the choroid in the eyes of many old people. Attention is called to this condition, one that may readily be overlooked, since it is perhaps more common than the two cases would seem to indicate. It is brought out while illuminating the fundus as much as possible, at the same time leaving the affected part of the iris in comparative darkness. As only three weeks had elapsed since the extraction, the time was too short for any extensive atrophy to occur. The pigment of the iris was not rubbed off by the lens in its passage through the pupil.

HOWARD F. HANSELL,
Clerk of Section.

---

### LUPUS VULGARIS IN THE WIFE AND DAUGHTER OF A TUBERCULOUS SUBJECT.

Dr. J. McF. Winfield (of Brooklyn) reports these cases in a paper presented at last meeting of the American Medical Association (*Jour. Am. Med. Assn.*, December 12, 1896):

The husband and father died of tuberculosis twelve years before. During his illness he was extremely uncleanly, wiping his mouth after expectoration upon the family towel and expectorating upon the floor by preference.

A small scab was rubbed off the wife's nose in using the towel. This was the starting point of a lupus patch covering the nose.

The child's face became extensively involved during the early years of its life, having first shown the disease before the father's death.

He thinks the infection of mother and child was easy through contact with the towels and floor contaminated by the husband and father.

